# Complete Ectopia Cordis: A Case Report and Literature Review

**DOI:** 10.1155/2017/1858621

**Published:** 2017-04-19

**Authors:** Simon Pius, Halima Abubakar Ibrahim, Mustapha Bello, Mohammed Bashir Tahir

**Affiliations:** ^1^Department of Paediatrics, University of Maiduguri Teaching Hospital, Maiduguri, Nigeria; ^2^Department of Surgery, University of Maiduguri Teaching Hospital, Maiduguri, Nigeria

## Abstract

Ectopia cordis is a congenital heart exposure defined as complete or partial protrusion of heart through ventral defect in the thoracoabdominal wall alone or with other viscera in cases of pentalogy of Cantrell. This condition was first described by Haller et al. in 1706; since then many advances have been made. Diagnosis of ectopia cordis is done prenatally in well-equipped health facility by antenatal ultrasound scan so that early diagnosis and management plan can be initiated. The index case was delivered to uneducated rural family and admitted at 3 days of life and survived for seven days, even though most literatures state that majority died within four days even with surgery. So, in view of this, we presented this case report to deliberately draw the attention of paediatrician/obstetrician to the fact that even though this condition is rare, proactive search and diagnosis should be made and early treatment should be instituted, so that such a child may be salvaged.

## 1. Introduction

Ectopia cordis (EC) is a rare congenital cardiac malformation defined as a defect in the anterior chest wall and abdominal wall with abnormal placement of the heart outside the thoracic cavity with associated defect in the parietal pericardium diaphragm, sternum, and in most cases cardiac malformations [1]. Ectopia cordis is also defined as complete or partial displacement of heart outside the thoracic cavity. Ectopia cordis was first observed 5000 years ago and the term ectopia cordis was first described by Haller et al. in 1706 [2]. It is generally a sporadic malformation, with reports linking it to chromosomal abnormalities like trisomy 18, Turner syndrome, 46,XX, and 17q+. The occurrence prevalence is estimated to be between 5.5 and 7.9/million live births [3–7].

Despite advances in neonatal cardiac surgery, complete thoracic/thoracoabdominal ectopia cordis remains a surgical challenge with only few long-term survivors [8–10]. We present a case report and review of literature and also draw the attention of neonatologist and other stakeholders in care of such patient by highlighting that even though this case is rare and prognosis following surgery is poor, this condition does occur and when identified early surgical intervention may make a difference even in poor-resources setting like ours.

## 2. Presentation of Case

B. F. M., an 11-day-old female neonate, admitted to the Special Care Baby Unit (SCBU) of University of Maiduguri Teaching Hospital, Maiduguri, Northeastern Nigeria, as referral case from Level II Hospital (secondary health facility) with complaints of protrusion of heart through the anterior chest wall defect at birth. There was no history of bleeding or discharge from the site. There was no history of difficulty in breathing or cyanosis. The child was fed with expressed goat milk via feeding bottle as she could not be placed on direct breast-feeding due to her condition. There was no history of other malformations or defect in other parts of the body and there was no family history of congenital defect. There was no maternal history of ingestion of unprescribed medications, use of illicit drugs, cigarette smoking, or alcohol abuse. The mother also had no history of chronic ill health. Their source potable water is from cement well.

Baby was delivered at home and attended to by traditional birth attendant without complications and she cried well at birth but with heart beating outside the chest wall and cord care with warm compression using wet rag and salt. Baby was the 3rd born of 3 children in a monogamous consanguineous marriage. Father is a 36-year-old rural subsistent farmer; both parents had no formal education.

Examination finding at admission revealed an acutely ill-looking neonate who was globally pink and she was febrile with temperature of 37.7°C and she was anicteric and not dehydrated. She weighed 3.2 kilograms; length was 47 cm and head circumference was 33 cm.

**Figure 1:**
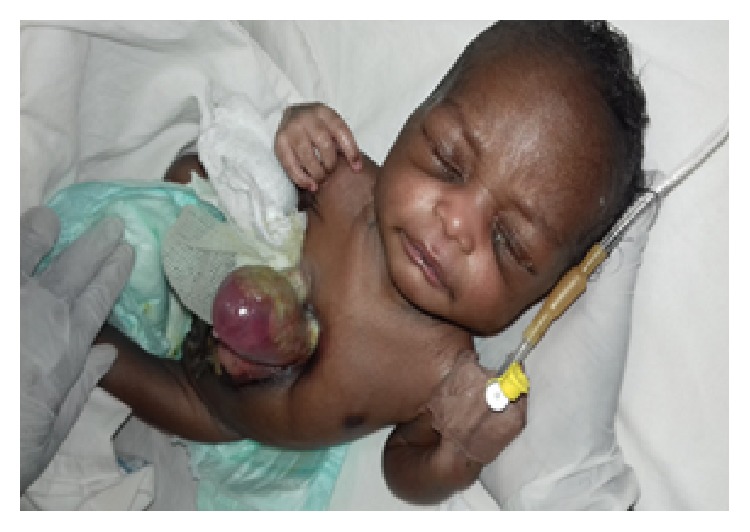
Ectopia cordis photograph from above.

**Figure 2:**
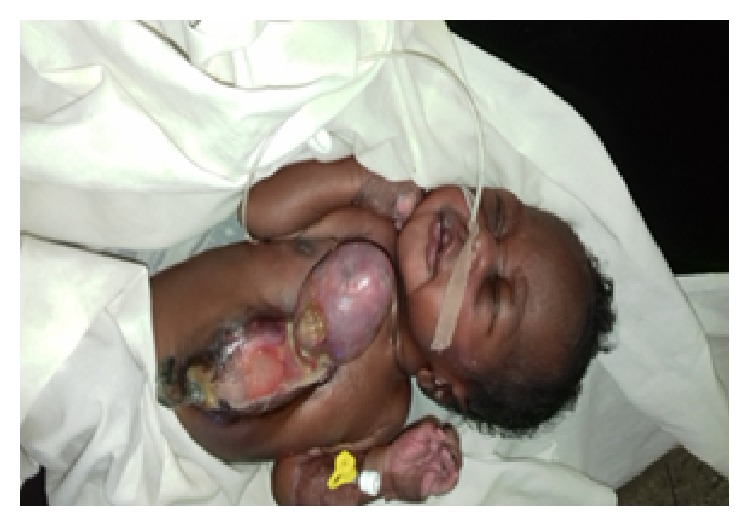
Ectopia cordis photograph with anterior abdominal wall defect.

*Diseased Organ.* There was a midline anterior chest wall defect measuring 6 by 4 cm in the craniocaudal and horizontal diameters extending in between the nipples and there was an ulcer extending from the defect to the umbilical stump. The whole of the heart with irregular contour was outside the thoracic cavity leaving only the major vessels in the chest wall as seen in Figure 1. There were nodular masses popping out on the heart wall firm in nature, not pulsating, and no obvious coronary vessels were seen on the cardiac wall as seen Figure 2.

The neonate was dyspnoeic and tachypnoeic respiratory rate was 98 CPM; there was good air entry. Heart rate was 162 BPM; heart sound was 1st and 2nd only; there was no murmur heard. Abdominal examination was unremarkable with well-formed female external genitalia. She was conscious and alert, anterior fontanelle was patent, no other midline defects were identified, and primitive reflexes were normal with good tones.

Working diagnosis of ectopia cordis to rule out pentalogy of Cantrell with presumed sepsis was made. Packed cell volume was 51%, WBC = 7.4 × 10^9^/L, neutrophil = 56, lymphocyte = 40, basophil = 2, and monocyte = 2. Platelets = 400 × 10^9^/L. E is electrolytes, U is urea, and C is creatinine; Na^+^ = 136 mmol/L, K^+^ = 3.4 mmol/L, Cl^−^ = 98 mmol/L, HCO_3_^−^ = 17 mmol/L, urea = 9.8 mmol/L, creatinine = 227 mmol/L, and Ca^+^ = 2.1 mml/L. Wound swab microscopy culture and sensitivity and blood culture yielded no bacterial growth.

**Figure 3:**
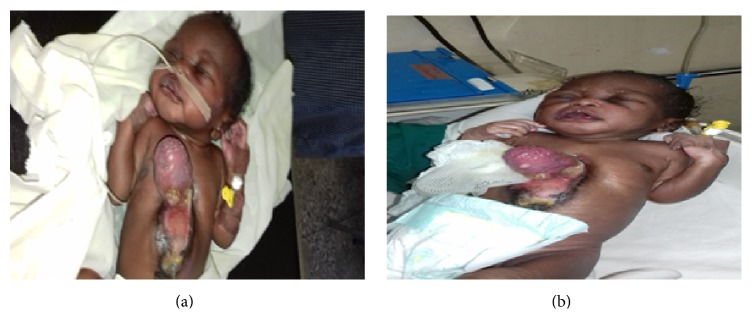
Ectopia cordis photograph in a plain view.

Patient was commenced on cefuroxime, gentamycin, and metronidazole. Surgical consultation with cardiothoracic surgery was done at admission, while the protruding heart was covered with antibiotic impregnated (Sofra-Tulle) gauze as seen in Figures 3(a) and 3(b). On the 4th day of admission, the child was stable, had nasogastric tube passed, and fed expressed breast milk at 3 ml every 2 hours; on this same day, the cardiothoracic centre at University of Nigeria Teaching Hospital, Enugu, approved the transfer of the child with surgical fee of three million naira which was to be provided by a donor. By 5th day, patient had tolerated 12 ml of expressed breast milk and intravenous fluid was discontinued. The child condition had deteriorated from 9th day while awaiting the transfer to the cardiothoracic centre in sister institution, when the heart beat became irregular and haemorrhagic while she was on antibiotics. By the time arrangement had been concluded for her to undergo cardiothoracic surgery, it was too late as the child condition had deteriorated and patient died on the 11th day at 3.30 AM.

## 3. Discussion

Ectopia cordis (EC), a congenital extrusion of heart through a defect on the anterior chest wall, currently has no known aetiology and most cases are sporadic [1]. The development of the ventral body wall begins by eighth day of the embryonic life with differentiation and proliferation of mesoderm followed by its literal migration. The heart originally develops in the cephalic location and reaches its definitive position by the lateral folding and ventral flexing of the embryo at about 16th-17th day of embryogenesis [2]. Midline fusion and formation of the thoracic and abdominal cavities are complete by the 9th embryonic week [11, 12].

Complete or incomplete failure of midline fusion at this stage results in disorders varying from isolated EC to complete ventral evisceration. Genesis of EC has not been fully explained, although several theories have been offered. Popular theories are early rupture of the chorion and/or yolk sac and amniotic band syndrome [2, 13–15]. The amniotic rupture theory states that, during early embryonic development, the amnion surrounding the embryo ruptures, and stringy, sticky, fibrous bands of amnion become entangled with forming embryo and cause a disruption in the developing parts of the foetus which may lead to various deformities like EC, midline sternal cleft, frontonasal dysgenesis, midfacial cleft, limb deformities, among others.

The spectrum of defects corresponds to the timing of rupture. The findings in the literature suggest that rupture in the third week of gestation causes an arrest of cardiac descent which may be the cause of EC. Ectopia cordis with amniotic bands appears to be distinct from isolated EC. This suggests different aetiologies for ectopia cordis; such aetiologies like exposure to intrauterine drug use have been documented in literatures [13–17]. The heart may be partially or completely outside thorax as described by Engum et al. [1, 13, 18] and, depending on the location of the heart, it is classified into different types, cervical (5%), cervicothoracic and thoracic (65%), thoracoabdominal (20%) to which our own belongs to, and abdominal (10%) [2, 19]. In partial EC, the heart can often be seen to pulsate through the skin, while in complete thoracic EC the naked heart is displaced outside the thoracic cavity without pericardial coverage [8, 17]. The thoracoabdominal type is regarded as distinct syndrome known as pentalogy of Cantrell. It consists of five associated anomalies: distal sternum defect, midline supraumbilical abdominal wall defect, ventral diaphragmatic hernia, defect in the epical pericardium and free communication into the peritoneal cavity, and congenital intracardiac defect [20].

The various clinical types of EC have different prognosis. Cervical and thoracic ectopia is usually fatal within days, because the heart is exposed and malformed; however, our case had thoracoabdominal ectopia cordis. Abdominal ectopia cordis carries a better prognosis, probably because intracardiac abnormalities are rarer and the absence of omphalocele reduces the morbidity and mortality [21]. Ectopia cordis is frequently associated with other congenital defects involving multiple organ system, and as much as 80.2% have associated intracardiac defects which include ventricular septal defect (VSD, 100%), atrial septal defect (ASD, 53%), Tetralogy of Fallot (TOF, 20%), left ventricular diverticulum (LVD, 20%), and pulmonary hypoplasia; even though in our case there was no murmur identified, we could not say there was no intracardiac defect as we were unable to do echocardiogram on the patient before demise [8, 9, 21].

The syndromic form known as pentalogy of Cantrell is extremely rare and usually incompatible with life; because of this, its exact incidence is not found in literature. In this condition, the heart is uncovered in 41%, covered with serous membrane in 31%, and covered with skin in 27% in a particular case series [22–24]. In our case, which is thoracoabdominal ectopia cordis, the heart was uncovered and the defect extended down to the umbilical region; though no other abdominal viscera were exposed.

On the bases of embryological development, this syndrome is classified into two groups. The first group arises as a result of developmental failure of a segment of the mesoderm and comprises three of the defects, which include diaphragmatic defect (which results from total or partial failure of the transverse septum to develop), pericardial defect (which is closely related to faulty development of the transverse septum), and intracardiac lesions (which is the result of faulty development of the epimyocardium, which is derived from the splanchnic mesoderm) [8, 22, 24].

The second group includes the sternal and abdominal wall defect and appears to arise due to failure of migration of the paired primordial structures. Many variants of Cantrell's pentalogy have been described according to postulated embryological development of these defects [8, 25, 26]. This includes the exact diagnosis, with five defects present, the probable diagnosis, with four defects present, and lastly incomplete diagnosis, with combination in the defects. The occurrence of congenital intracardiac anomalies is a constant element of this syndrome; with the increasing use of antenatal diagnostic tools, these anomalies can be diagnosed before birth [8, 24]. The other associated malformations such as cyllosomas (another name for limb-body wall complex) are defined as anomaly consisting of two of the following three fetal anomalies: (a) thoracoabdominoschisis or abdominoschisis, (b) limb defect, (c) craniofacial defects: cleft lip/palate, encephalocele, exencephaly, among others [27].

The prenatal diagnosis of ectopia cordis is carried out using ultrasound, which allows visualization of the heart outside the thoracic cavity. The diagnosis as reported by Bick et al. [28] and Tongsong et al. [29] has been made as early as between 9 and 11 weeks of gestation, respectively. The use of three-dimensional ultrasound and its combination with Doppler allows for a more accurate early diagnosis. Magnetic resonance imaging is also becoming commonplace in prenatal evaluation to document and plan for management of complicated congenital anomalies. In the index case, the mother is from remote desert rural farming community where robust health facility for antenatal care to enable such a prenatal investigation for early detection of this diagnosis does not exist. In the absence of such an antenatal care, the delivery took place at home and the baby was attended to by unskilled traditional birth attendants, which contributed to late diagnosis of the condition. The patient presented to our facility on third day of life with the protruding heart covered with dirty rag; in addition, the newborn subsequently developed fever and respiratory difficulty which might have contributed to poor outcome.

While ectopia cordis is generally considered to be an isolated, sporadic malformation, there have been a number of reports linking it to chromosomal abnormalities. Reported karyotype abnormalities include trisomy 18, Turner syndrome, 46,XX, and 17q+ [30]. Carmi and Boughman described other defects observed to be associated with this malformation which include cleft lip with or without cleft palate and encephalocele as part of the ventral midline defect in the spectrum of Cantrell's pentalogy [31]. Newborn with these complex and life-threatening anomalies require intensive care right from birth because of rapid deterioration in their condition. The immediate management approach requires resuscitation and coverage of the exposed heart and viscera with saline-soaked gauze pads wrappings to prevent desiccation and heat loss [32]. In our case, though patient presented on third day, we resuscitated and covered exposed heart with Sofra-Tulle gauze in addition to antibiotic administration; patient had spent eleven days on admission awaiting definitive cardiothoracic surgery contrary to what has been documented in literature [30, 32].

The ultimate management of the condition is surgical correction. The first attempted repair of ectopia cordis was performed in 1925 by Cutler and Wilens, [33] and Koop (1975) achieved the first successful repair of thoracic ectopia cordis in two stages [34]. Amato et al. reported a successful single-stage repair of thoracic ectopia cordis in 1995 [35]. During surgical closure, in most of the cases, the thoracic cavity is small with little mediastinal space for the heart. Attempt to close the chest wall often results in haemodynamic embarrassment secondary to kicking of the great vessels possibly due to their long length and abnormal course or compression of the heart [31]. The steps usually taken for the treatment (all variety) are (1) closure of the chest wall defect (either by doing primary chest wall closures or by using bone/cartilage as tissue graft or artificial prosthesis like acrylic plaques and Marlex mesh), (2) closure of the sternal defect, (3) repair of the associated defect such as omphalocele, though in our case there was no exposure of intra-abdominal organs, (4) placement of the heart into the thorax, and (5) repair of the intracardiac defect [2, 31, 32].

The staged repair with the initial intervention is aimed at providing soft tissue coverage of the heart. If primary approximation with skin flaps cannot be achieved or if there is any haemodynamic compromise, then a split-thickness skin graft, cadaveric skin graft, or prosthetic materials can be sutured to the skin edges. The defect can then be reduced slowly over the ensuing weeks [9, 27, 36]. The timing for palliation of haemodynamically significant intracardiac defects is unclear; however, report by Morales et al. [9] suggested that the placement of a Blalock-Taussig shunt or a pulmonary artery band should be deferred for a few weeks until the haemodynamic effects of chest coverage have stabilized.

The reduction of the heart into the thoracic cavity is carried out at a subsequent stage [8, 33]. Simple stable intracardiac defects like ASD, VSD, LVD, and TOF can be repaired at this time. Reduction of the heart into a small thoracic cavity in the neonatal period often produces compression and kinking of the great vessels usually leads to low cardiac output. However, it has been suggested that it is worthwhile making attempt at placing the heart even partially within the thoracic cavity at the first stage. This will make subsequent procedures easier and also avoid the obvious physical deformity. The heart can be returned to the left or right depending on which direction the apex points [8, 27, 34]. Chest wall reconstruction is carried out at a later date in collaboration with plastic surgeon. In the index case, there was no murmur and there was no diaphragmatic hernia; however, the heart was exposed with no pericardial covering and the thoracoabdominal defect extended to umbilical region.

In literature, most of the patients died within 4 days following surgical intervention whether the case was isolated ectopia cordis or pentalogy of Cantrell, while our patient, a case of isolated ectopia cordis, survived for 10 days before she died; to our minds, we failed the child; we failed to give her a chance to live despite giving us enough time to salvage her.

## 4. Conclusion

In conclusion, this index case of EC survived for 10 days, and if aggressive surgical procedures were carried out without delay in the believe that this may enhance survival, probably this child could have lived. Ectopia cordis is a rare congenital malformation with a poor prognosis. Antenatal ultrasound scan is of great value in the prenatal assessment of this defect. The defect should be precisely located and its classification should be accurately determined; in view of the different types of ectopia cordis, even if the overall prognosis is not rewarding, their surgical approach varies as well as the ultimate outcome. The health care delivery in developing countries is poorly funded as consequence of poor policy formulation and implementation, thereby leaving it ill equipped, which might have contributed to the outcome of the index patients; otherwise this patient probably should not have died if our centre had facility for cardiac surgery. Therefore, this case report is another lesson to ponder upon so that the next child with ectopia cordis should not suffer the same fate.
